# Genetic uniformity, geographical spread and anthropogenic habitat modifications of lymnaeid vectors found in a One Health initiative in the highest human fascioliasis hyperendemic of the Bolivian Altiplano

**DOI:** 10.1186/s13071-020-04045-x

**Published:** 2020-04-06

**Authors:** M. Dolores Bargues, Patricio Artigas, Rene Angles, David Osca, Pamela Duran, Paola Buchon, R. Karina Gonzales-Pomar, Julio Pinto-Mendieta, Santiago Mas-Coma

**Affiliations:** 1 Departamento de Parasitología, Facultad de Farmacia, Universidad de Valencia, Av. Vicente Andrés Estellés s/n, Burjassot, 46100 Valencia, Spain; 2 Cátedra de Parasitología, Facultad de Medicina, Universidad Mayor de San Andrés (UMSA), Av. Saavedra, Miraflores, La Paz, Bolivia; 3 Unidad de Limnología, Instituto de Ecología, Universidad Mayor de San Andrés (UMSA), Campus Universitario de Cota Cota, Calle 27, La Paz, Bolivia

**Keywords:** Human fascioliasis, Lymnaeids, *Galba truncatula*, rDNA, mtDNA, Geographical spread, Habitat modifications, One Health, Northern Bolivian Altiplano

## Abstract

**Background:**

Fascioliasis is a snail-borne zoonotic trematodiasis emerging due to climate changes, anthropogenic environment modifications, and livestock movements. Many areas where *Fasciola hepatica* is endemic in humans have been described in Latin America altitude areas. Highest prevalences and intensities were reported from four provinces of the northern Bolivian Altiplano, where preventive chemotherapy is ongoing. New strategies are now incorporated to decrease infection/re-infection risk, assessment of human infection sources to enable efficient prevention measures, and additionally a One Health initiative in a selected zone. Subsequent extension of these pilot interventions to the remaining Altiplano is key.

**Methods:**

To verify reproducibility throughout, 133 specimens from 25 lymnaeid populations representative of the whole Altiplano, and 11 used for population dynamics studies, were analyzed by rDNA ITS2 and ITS1 and mtDNA *cox*1 and *16S* sequencing to assess their classification, variability and geographical spread.

**Results:**

Lymnaeid populations proved to belong to a monomorphic group, *Galba truncatula*. Only a single *cox*1 mutation was found in a local population. Two *cox*1 haplotypes were new. Comparisons of transmission foci data from the 1990’s with those of 2018 demonstrated an endemic area expansion. Altitudinal, northward and southward expansions suggest movements of livestock transporting *G. truncatula* snails, with increasing temperatures transforming previously unsuitable habitats into suitable transmission areas. Transmission foci appear to be stable when compared to past field observations, except for those modified by human activities, including construction of new roads or control measures undertaken in relation to fascioliasis.

**Conclusions:**

For a One Health initiative, the control of only one *Fasciola* species and snail vector species simplifies efforts because of the lower transmission complexity. Vector monomorphism suggests uniformity of vector population responses after control measure implementation. Hyperendemic area outer boundary instability suggests a climate change impact. All populations outside previously known boundaries were close to villages, human dwellings and/or schools, and should therefore be considered during disease control planning. The remarkable southward expansion implies that a fifth province, Aroma, should now be included within preventive chemotherapy programmes. This study highlights the need for lymnaeid molecular identification, transmission foci stability monitoring, and potential vector spread assessment.
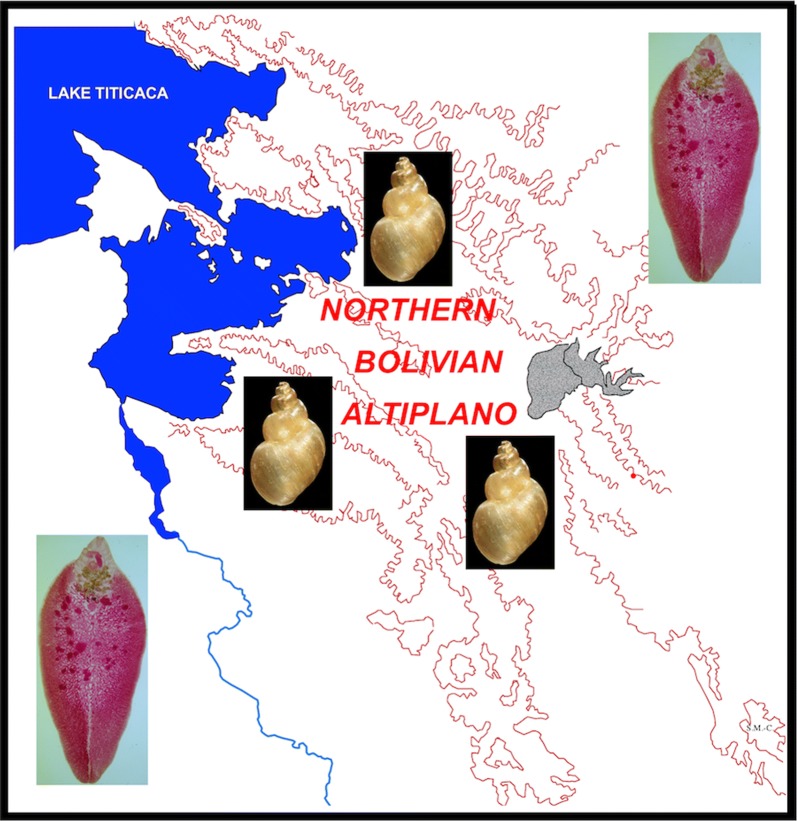

## Background

Fascioliasis is a snail vector-borne zoonotic trematodiasis which may be highly pathogenic in humans [1], both in the initial short acute phase during the tissular migration of the infective small juvenile flukes and in the subsequent chronic phase during the long-term infection by the large adult flukes in the biliary canals [2–6]. Despite of this, human fascioliasis was only considered of secondary public health importance up until 1990 [7].

Since 1990, human fascioliasis endemic areas began to be described in certain countries and an increasing number of human cases were reported [8]. The effects caused by this disease become more problematic due to the immunosuppression induced by the liver fluke in the chronic and advanced chronic periods of the disease [9], this is when infected subjects are usually diagnosed in human fascioliasis endemic areas. Immunosuppression underlies the usual coinfections of *Fasciola*-infected subjects with other pathogenic protozoans (e.g. *Giardia intestinalis*, *Cryptosporidium* sp.) and helminths (e.g. *Ascaris lumbricoides*, *Trichuris trichiura*), thereby pronouncedly increasing morbidity in human endemic areas [10–13].

Increasing infection rates have been linked to the impact of climate change, both regarding animal fascioliasis [14] and more recently also human fascioliasis [15], similarly as it has been observed for other snail-borne trematodiases [16]. This new scenario has been considered of sufficient impact as to include fascioliasis within the group of food-borne trematodiases among the list of main neglected tropical diseases by the World Health Organization since the 1990s [17]. This scenario is characterized by a large heterogeneity and complexity in transmission patterns and epidemiological situations, characterized by multidisciplinary factors such as: environmental conditions; climate characteristics; lymnaeid species present; vector-specificity of the *Fasciola* species and their biogeography; livestock species present; local traditions of livestock management; sylvatic reservoir fauna; behaviour and social traditions and diet of the human communities; education and hygiene; among many others [8, 18].

Two *Fasciola* species, *F. hepatica* and *F. gigantica*, cause human fascioliasis, but only *F. hepatica* is present in the Americas [18]. Many human fascioliasis endemic areas have been described in the Americas [8, 18], from Mexico [19] to several countries in South America, such as Peru including Altiplano [12] and valleys [13, 20–22], Chile [23, 24] and Argentina [25–27]. Other countries including Venezuela [28], Colombia and Ecuador [29] have also reported human infections. It should be emphasized that such human endemic areas are found in the highlands of these Andean countries, where liver fluke transmission is enhanced in the high altitude environments [30].

**Fig. 1:**
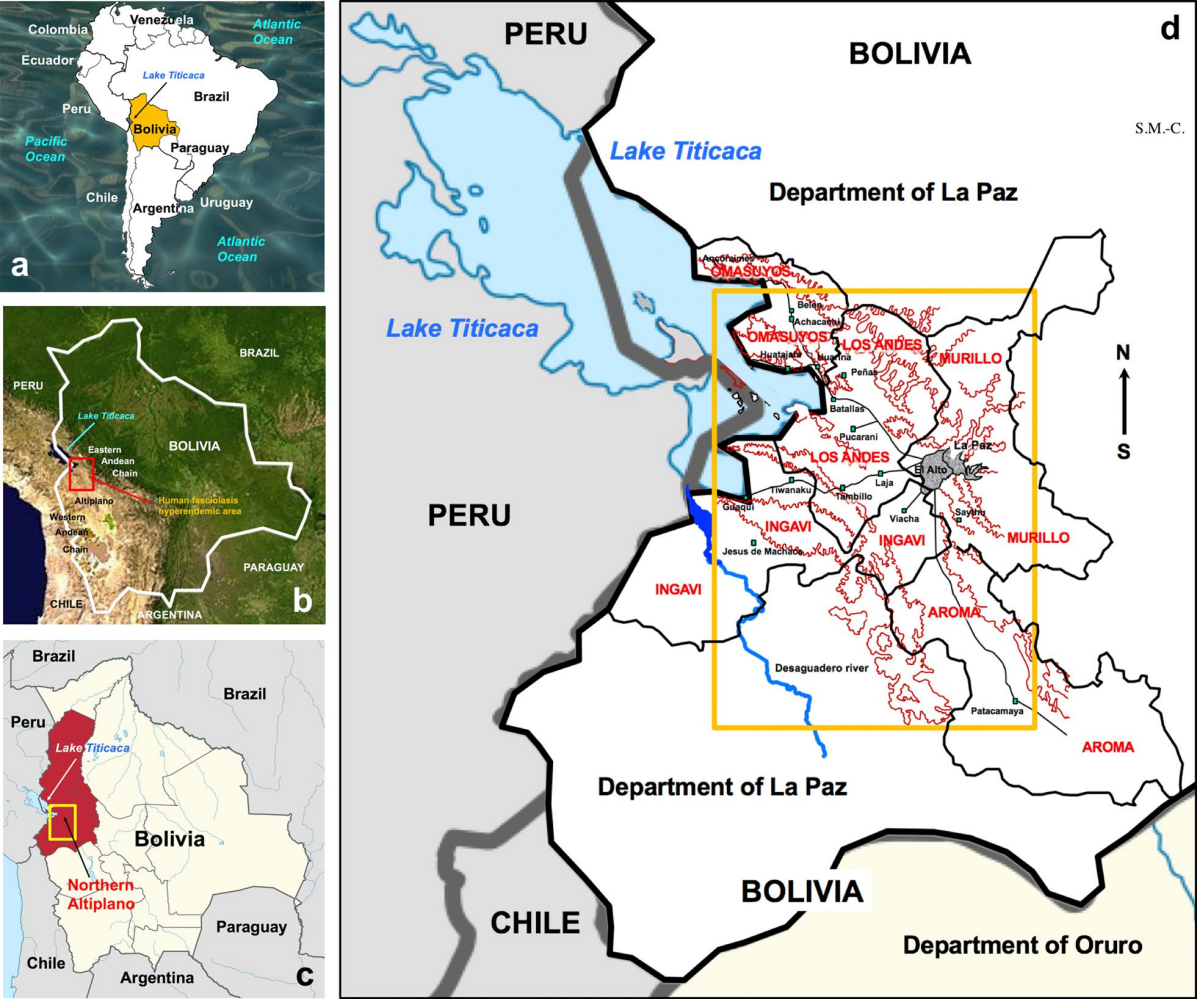
Location of the northern Bolivian Altiplano human fascioliasis hyperendemic area. **a** Map showing the location of Bolivia in South America. **b** Topographic map showing the location of the endemic area in the very high-altitude region of the northern Altiplano close to Lake Titicaca and eastern Andean chain. **c** Political map showing the endemic area inside the Bolivian Department of La Paz. **d** Political and geographical map showing the endemic area throughout the corridors and zones between Lake Titicaca and the Bolivian capital of La Paz, dispersed within the five provinces of Omasuyos, Los Andes, Murillo, Ingavi and Aroma of the Department of La Paz. Background for **b** from composed satellite map of South America orthographic projection by NASA (full resolution of 1215 × 1712 pixels; public domain) *via* Wikimedia Commons. Hand-made drawing for **d** created using Microsoft® PowerPoint for Mac v. 16.25. Original SM-C

Among all South American countries, Bolivia has reported the highest human prevalences and intensities ever described, namely in the northern Bolivian Altiplano in areas of very high altitude (3820–4100 m) between Lake Titicaca and the La Paz Valley (Fig. 1) [10, 11, 31–34]. It was in this area where the WHO launched a pilot study which demonstrated that triclabendazole is effective for preventive chemotherapy [22, 35]. After this successful pilot initiative carried out between 2007 and 2008, WHO launched a long-term preventive chemotherapy strategy by means of annual mass drug administration with a single treatment of Egaten® (triclabendazole for human use) donated by Novartis Pharma AG (Basel, Switzerland). The purpose of this preventive chemotherapy strategy was to decrease morbidity, mainly in schoolchildren, the most affected age group [33]. This strategy has been locally implemented by the Bolivian Ministry of Health and in the field by the Servicio Departamental de Salud de La Paz (SEDES La Paz) for the last ten years.

However, livestock, including cattle, sheep, pigs and donkeys are crucial for community sustainability throughout this rural endemic area because plant cultures are rarely practiced due to the problem posed by the extreme conditions of the very high altitude. Thus, liver fluke infection of these domestic animals assures fascioliasis endemicity and consequent human infection and re-infection risks [36], with continued infections observed during local inter-annual monitoring. Therefore, two further strategies are now being incorporated to this preventive chemotherapy initiative to decrease the risk of human infection. The first is the assessment of human infection sources and awareness/education [37]. The second is the implementation of One Health initiatives in relation to zoonotic transmission [38], an important factor in controlling human fascioliasis [39].

The One Health pilot initiative has been launched in a small selected part of the human fascioliasis hyperendemic area of the northern Bolivian Altiplano. This was to enable the detailed monitoring of the five factors linked to transmission: (i) the lymnaeid snail vector populations; (ii) the animal reservoirs; (iii) the environment and its changing trends including climate change and anthropogenic modifications; (iv) the human host; and (v) social, tradition and behavioural aspects.

In fascioliasis, lymnaeid snails play a crucial role in transmission and epidemiology. The aims of the present study include the classification, genetic variability and geographical spread of lymnaeids to assess whether the fascioliasis characteristics are similar and stable throughout the whole endemic area. Localities were selected to furnish a representative picture of the whole endemic area and to analyze whether changes have occurred in given transmission foci. Outside the past endemic area, prospections were made where physiography a priori would allow snails to expand because of the similar ecological characteristics and presence of livestock. Sequencing of complete DNA markers is therefore used. For the analyses of the geographical spread and modifications of the transmission foci occurred over time, recent results are compared with results of studies implemented during the 1990s [34]. It should be highlighted that the Altiplano is the only human fascioliasis hyperendemic area where a broad multidisciplinary knowledge on the disease was obtained in the past. This allows for a past/present comparison to assess how a human endemic area evolves over time.

## Methods

### Lymnaeid studies

**Fig. 2:**
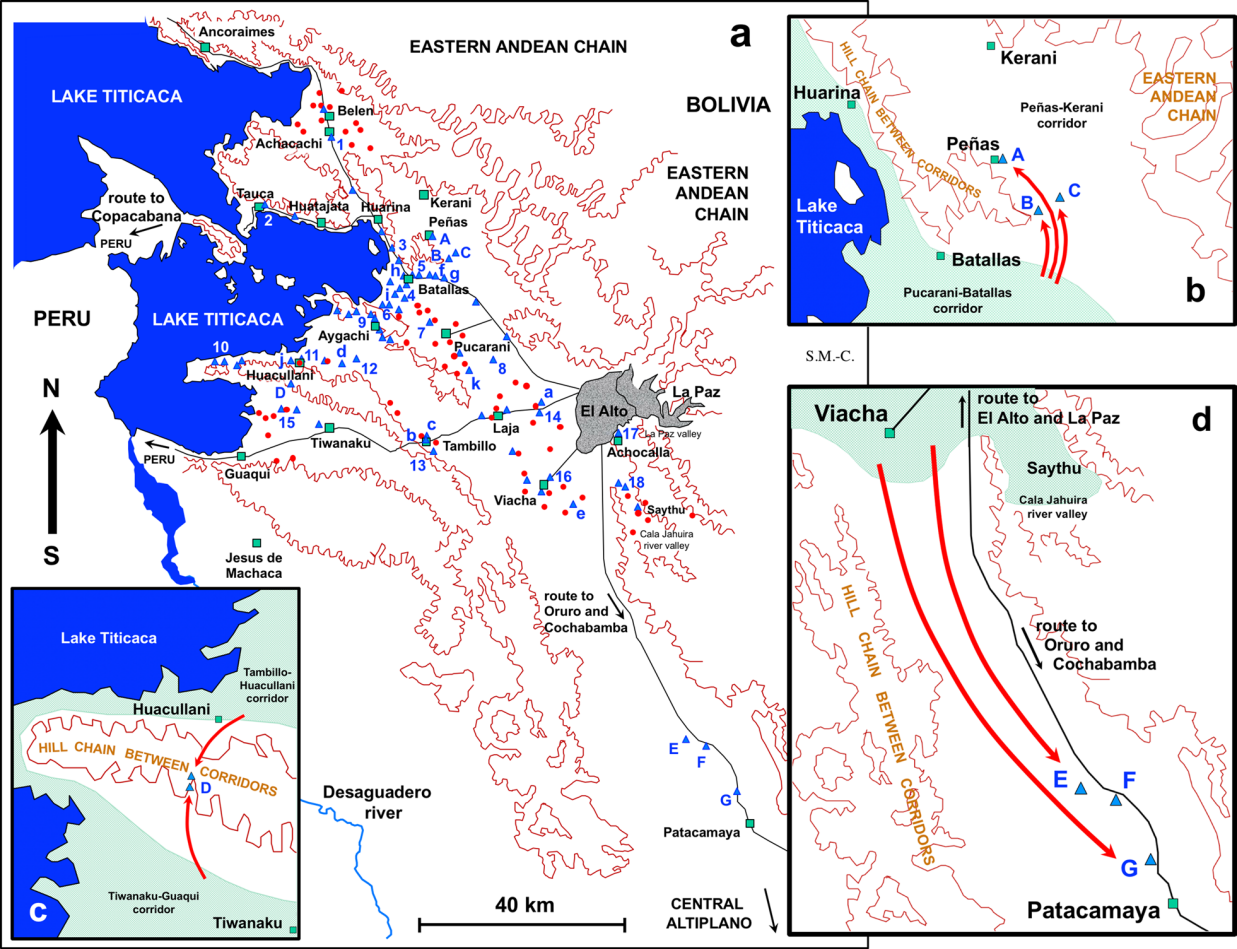
Northern Bolivian Altiplano human fascioliasis hyperendemic area. **a** Map showing the location of the lymnaeid vector populations studied. **b** Magnified map showing northward lymnaeid population spread into the Peñas-Kerani corridor. **c** Magnified map showing altitudinal lymnaeid population spread into the hill chain between the Tambillo-Huacullani corridor and the Tiwanaku-Guaqui corridor. **d** Magnified map showing southward lymnaeid population spread up to the Patacamaya zone. Hand-made drawing created using Microsoft® PowerPoint for Mac v. 16.25. Original SM-C. *Key*: Blue triangles, freshwater habitats presenting lymnaeid populations; red circles, localities where cattle was proved to be infected by the liver fluke in previous studies; green squares, human villages; grey shaded areas, large cities of La Paz and El Alto; brown outline, mountainous areas delimiting flatlands and corridors; green-shaded parts in **b**, **c** and **d**, zones of altitudes suitable for lymnaeid existence in the past; numbers/letters correspond to lymnaeid vector populations studied (see Table 1) inside/outside the past established boundaries of the endemic area [34]

The presence of lymnaeid populations in this endemic area has been assessed by traditional malacological searching and sampling methods [40–42]. Lymnaeid snails were collected in multiple localities of the fascioliasis endemic area of the northern Bolivian Altiplano (Figs. 1, 2).

#### Snail collection

Field studies of snails were made for two research purposes:

(i) to assess the presence or absence of lymnaeids in freshwater habitats and their geographical distribution. Studies included sites surveyed in the past, independently on whether they presented lymnaeids or not [34]. Freshwater habitats not analyzed in the past were also surveyed. Snail collection was made between 10:00 h and 13:00 h. A minimum of four people participated in the surveys at each collection site. Both the water margins and surrounding humid mud zones were surveyed. Lymnaeids were collected and initially morphologically identified by their small, smooth and dextral conical shell and their pair of triangular tentacles with darkly pigmented eyes at their bases [43].

(ii) for the follow-up of lymnaeid population dynamics in selected different transmission foci throughout a complete 12-month period and collecting of living specimens for subsequent experimental studies of their embryonic development, growth, fecundity and life span. Specimens from these foci were also used for DNA sequencing. Snail specimens from each freshwater collection were fixed in 96% ethanol for subsequent molecular analyses (Table 1).

**Table 1:** Geographical location, nuclear ribosomal ITS and mitochondrial DNA gene haplotype code identification for *Galba truncatula* populations studied from the northern Bolivian Altiplano human fascioliasis hyperendemic area

Code	Locality	Corridor/zone	Province	Latitude (S)	Longitude (W)	Altitude (m)	ITS2	ITS1	*cox*1	*16S*
							Acc. No.	Acc. No.	Acc. No.	Acc. No.
1	Achacachi (*n* = 4)	Zone of Achacachi	Omasuyos	16°03’51”	68°40’40”	3850	AJ272051	AJ272052	MN010644	HE610431
2	Tauca (*n* = 2)	Lake shore of Huatajata-Tauca	Omasuyos	16°10’33”	68°48’13”	3845	AJ272051	AJ272052	MN010644	HE610431
3	Cupamcara (*n* = 4)	Lake shore of Huarina	Los Andes	16°14’28”	68°34’28”	3835	AJ272051	AJ272052	MN010644	HE610431
4	Batallas canal (*n* = 6)	Corr. of Pucarani-Batallas	Los Andes	16°18’08”	68°32’12”	3850	AJ272051	AJ272052	MN010644	HE610431
5	Rio Karawisa (*n* = 6)	Corr. of Pucarani-Batallas	Los Andes	16°17’46”	68°31’01”	3872	AJ272051	AJ272052	MN010644	HE610431
6	Cutusuma (*n* = 2)	Corr. of Pucarani-Batallas	Los Andes	16°20’07”	68°33’52”	3844	AJ272051	AJ272052	MN010644	HE610431
7	Rio Sehuenca (*n* = 2)	Corr. of Pucarani-Batallas	Los Andes	16°23’04”	68°29’20”	3861	AJ272051	AJ272052	MN010644	HE610431
8	Corapata (*n* = 4)	Corr. of Pucarani-Batallas	Los Andes	16°25’51”	68°23’20”	3884	AJ272051	AJ272052	MN010644	HE610431
9	Aygachi (*n* = 4)	Corr. of Tambillo-Huacullani	Los Andes	16°23’16”	68°35’55”	3832	AJ272051	AJ272052	MN010644	HE610431
10	Zapana (*n* = 4)	Corr. of Tambillo-Huacullani	Ingavi	16°26’13”	68°52’49”	3847	AJ272051	AJ272052	MN010644	HE610431
11	Huacullani north (*n* = 6)	Corr. of Tambillo-Huacullani	Los Andes	16°26’27”	68°44’19”	3834	AJ272051	AJ272052	MN010644	HE610431
12	Lacaya (*n* = 4)	Corr. of Tambillo-Huacullani	Los Andes	16°26’27”	68°41’54”	3835	AJ272051	AJ272052	MN010644	HE610431
13	Tambillo stream (*n* = 4)	Corr. of Tambillo-Huacullani	Los Andes	16°34’29”	68°30’30”	3874	AJ272051	AJ272052	MN010644	HE610431
14	Kallutaca grassland (*n* = 4)	Corr. of Pucarani-Batallas	Los Andes	16°31’29”	68°18’22”	3906	AJ272051	AJ272052	MN010644	HE610431
15	Yanarico (*n* = 2)	Corr. of Tiwanaku-Guaqui	Ingavi	16°31’15”	68°45’51”	3838	AJ272051	AJ272052	MN010644	HE610431
16	Viacha (*n* = 4)	Zone of Viacha	Ingavi	16°37’58”	68°16’’47”	3872	AJ272051	AJ272052	MN010644	HE610431
17	Achocalla (*n* = 2)	Valley of La Paz	Murillo	16°34’43”	68°11’46”	3769	AJ272051	AJ272052	MN010644	HE610431
18	Tuni (*n* = 2)	Zone of Saythu	Murillo	16°40’21”	68°08’77”	3951	AJ272051	AJ272052	MN010644	HE610431
a	Kallutaca canal (*n* = 3)	Corr. of Pucarani-Batallas	Los Andes	16°31’30”	68°18’19”	3906	AJ272051	AJ272052	MN010644	HE610431
b	Tambillo inside village (*n* = 3)	Corr. of Tambillo-Huacullani	Los Andes	16°34’16”	68°30’29”	3865	AJ272051	AJ272052	MN010644	HE610431
c	Tambillo out of village (*n* = 3)	Corr. of Tambillo-Huacullani	Los Andes	16°34’12”	68°30’27”	3861	AJ272051	AJ272052	MN010644	HE610431
d	Quiripujo (*n* = 3)	Corr. of Tambillo-Huacullani	Los Andes	16°26’37”	68°39’55”	3840	AJ272051	AJ272052	MN010644	HE610431
e	Rio Achicala (*n* = 3)	Zone of Viacha	Ingavi	16°41’46”	68°16”22”	3866	AJ272051	AJ272052	MN010644	HE610431
f	Chirapaca 1 (*n* = 3)	Corr. of Pucarani-Batallas	Los Andes	16°17’56”	68°30”23”	3887	AJ272051	AJ272052	MN010644	HE610431
g	Chirapaca 2 (*n* = 3)	Corr. of Pucarani-Batallas	Los Andes	16°17’59”	68°29”55”	3893	AJ272051	AJ272052	MN010644	HE610431
h	Batallas slaughter. (*n* = 3)	Corr. of Pucarani-Batallas	Los Andes	16°17’45”	68°32’23”	3850	AJ272051	AJ272052	MN010644	HE610431
i	Chijipata Alto (*n* = 3)	Corr. of Pucarani-Batallas	Los Andes	16°18’37”	68°32’42”	3844	AJ272051	AJ272052	MN010644	HE610431
j	Huacullani west (*n* = 3)	Corr. of Tambillo-Huacullani	Los Andes	16°26’29”	68°44’36”	3837	AJ272051	AJ272052	MN010644	HE610431
k	Ancocagua (*n* = 3)	Corr. of Pucarani-Batallas	Los Andes	16°25’28”	68°27’23”	3853	AJ272051	AJ272052	MN010644	HE610431
A	Peñas (*n* = 6)	Corr. of Peñas-Kerani	Los Andes	16°13’52”	68°30’10”	3986	AJ272051	AJ272052	MN010644	HE610431
B	San Calixto (*n* = 4)	Corr. of Peñas-Kerani	Los Andes	16°16’07”	68°28’21”	3970	AJ272051	AJ272052	MN010644	HE610431
C	Suriquiña (*n* = 4)	Corr. of Peñas-Kerani	Los Andes	16°15’25”	68°27’31”	4001	AJ272051	AJ272052	MN010644	HE610431
D	Rosa Pata (*n* = 4)	Intercorridor hill chain^a^	Los Andes	16°28’41”	68°45’18”	3965	AJ272051	AJ272052	MN010644	HE610431
E	Challapata (*n* = 4)	Zone of Patacamaya	Aroma	17°05’15”	68°02’38”	3899	AJ272051	AJ272052	MN010644	HE610431
F	Ayo Ayo (*n* = 8)	Zone of Patacamaya	Aroma	17°05’43”	68°01’13”	3890	AJ272051	AJ272052	MN010644	HE610431
							AJ272051	AJ272052	MN010645	HE610431
G	Viscachani (*n* = 4)	Zone of Patacamaya	Aroma	17°10’39”	67°56’34”	3840	AJ272051	AJ272052	MN010644	HE610431

^a^Hill chain between corridors of Huacullani and Tiwanaku

*Notes*: See map in Fig. 2 for numbers or letters of lymnaeid vector population localities: numbers/letters correspond to populations inside/outside the past established borders of the endemic area [34]. No./letter in map, 1–18 indicates populations studied for geographical distribution assessment; a–k indicates populations used for population dynamics studies; A-G indicates new populations found outside the hitherto known boundaries of the endemic area. AJ272051, haplotype ITS2 H3; AJ272052, haplotype ITS1 HC; MN010644 and MN010645, new haplotypes *cox*1e and *cox*1f, respectively; HE610431, haplotype 16S-A; mtDNA haplotypes are preliminary due to incomplete gene sequences

*Abbreviations*: Acc. No., GenBank accession number; n, number of specimens sequenced; code, number or letter in the maps

Geographical coordinates were determined for each sampling site using GPS and afterwards mapped using Google Earth Pro 7.3.2.5776. Given the typically patchy distribution of human fascioliasis [34], dotting of the transmission foci including lymnaeid-inhabited freshwater bodies is made for the mapping illustration, according to geographical coordinates and following WHO methods for freshwater snail-borne diseases [44]. The geographical distribution of the lymnaeid populations assessed is shown in Fig. 2a.

#### Geographical surveys

A detailed geographical outline of the fascioliasis endemic area was established during the 1990s [34]. Past surveys on humans, cattle and lymnaeids demonstrated that this endemic area is completely isolated. Cattle were used as a marker because of the shorter lifespan of fasciolids in bovines [34, 45]. A total of 57 lymnaeid-inhabited freshwater sites, and around 14,000 specimens collected in different years and different seasons of the year, allowed for the delimitation of the geographical boundaries of the hyperendemic area (Fig. 2a) [34]. The boundaries proved to cover from the southern surroundings of the locality of Ancoraimes at the coast of Lake Titicaca, in the north, to a little southward from the locality of Viacha on the route from El Alto to Oruro, in the south. Longitudinally, the endemic area proved to extend from the valleys of La Paz and the River Cala Jahuira, in the east, to the Bolivian coast of Lake Titicaca in the west (Figs. 1, 2a) [34, 45]. This distribution concerned four provinces of the Department of La Paz (Fig. 1c, d); Los Andes, Ingavi, Omasuyos and Murillo (Fig. 2).

In the present study we describe the results of the 2018–2019 lymnaeid snail surveys made for two geographical purposes: (i) assessing presence/absence of lymnaeids in freshwater habitats located inside the whole endemic area to verify the stability of the patchy distribution established in the 1990s [34]; and (ii) analysis of freshwater habitats located in a wide perimeter outside the endemic boundaries established in the 1990s [34], to assess potential present and further spread of the disease occurred during the last 25 years. For both purposes, the same lymnaeid-inhabited water bodies studied in the past and other freshwater habitats in which lymnaeids were not found in the past, whether inside or outside the old endemic boundaries, were surveyed again.

### Molecular analysis

The complete nuclear ribosomal DNA (rDNA) ITS2 and ITS1 and fragments of the mitochondrial DNA (mtDNA) *16S* rDNA and *cox*1 were analyzed. These markers have been used previously to investigate the intraspecific variability of lymnaeid populations [46], in Bolivia [47], and in many countries of the Americas [48–50].

#### DNA extraction

Snails specimens for molecular analyses were transferred from 96% ethanol to 70% ethanol and then extractions were performed individually from the head-foot tissue of each snail using the phenol-chloroform method. Overall, 133 specimens randomly selected from many freshwater habitats, including 25 lymnaeid populations especially selected to be a representative sample covering the northern Bolivian Altiplano, plus other 11 lymnaeid habitats used for population dynamics studies and experimental purposes (see Table 1, Fig. 2), were suspended in 400 µl of lysis buffer (10 mM Tris-HCl, pH 8.0, 100 mM EDTA, 100 mM NaCl, 1% sodium dodecyl sulfate (SDS) containing 500 µg/ml Proteinase K (Promega, Madison, WI, USA) and digested for 2 h at 55 °C with alternate shaking every 15 min. The procedure steps were performed according to methods outlined previously [48, 49, 51]. The pellet was dried and resuspended in 30 µl sterile TE buffer (pH 8.0) and this suspension was stored at − 20 °C until use.

#### Analysis of the nuclear rDNA and mtDNA markers

The four DNA markers were amplified using previously described primers [49, 52–54]. Amplification procedures and thermal cycler conditions were previously described [43]. PCR products were purified using the Ultra Clean™ PCR Clean-up DNA Purification System (MoBio, Solana Beach, CA, USA) according to the manufacturer’s protocol and resuspended in 50 μl of 10 mM TE buffer (pH 7.6). The final DNA concentration was determined using an Eppendorf BioPhotometer (Eppendorf, Hamburg, Germany). Amplicons were Sanger sequenced in both strands on an Applied Biosystems 3730 DNA analyzer (Applied Biosystems, Foster City, CA, USA) using dilutions of the original PCR primers.

#### Sequence analysis

Sequences were edited and assembled by using Sequencher v5.4.6. (Gene Codes Co.) and aligned using ClustalW2 [55] in MEGA 6.0.6 [56], using default settings. Minor corrections for a better fit of nucleotide or indel correspondences in alignments were made in the cases of the ITS spacers. Sequences were identified using the BLASTn (National Centre for Biotechnology information website-http://www.ncbi.nlm.nih.gov/BLAST). Comparative sequence analyses and haplotype identification of lymnaeids were made in alignments using all available ribosomal and mitochondrial sequence data downloaded from GenBank.

#### DNA haplotype nomenclature

The haplotype (H) terminology used for the sequences obtained follows the standard nomenclature proposed for lymnaeid snails previously described [18, 46]. For each DNA marker, a haplotype code includes all identical sequences. Haplotype codes were only definitive in the case of complete sequences. When dealing with fragments or incomplete sequences, haplotype codes are provisional.

## Results

### Molecular characterization of lymnaeids

**Fig. 3:**
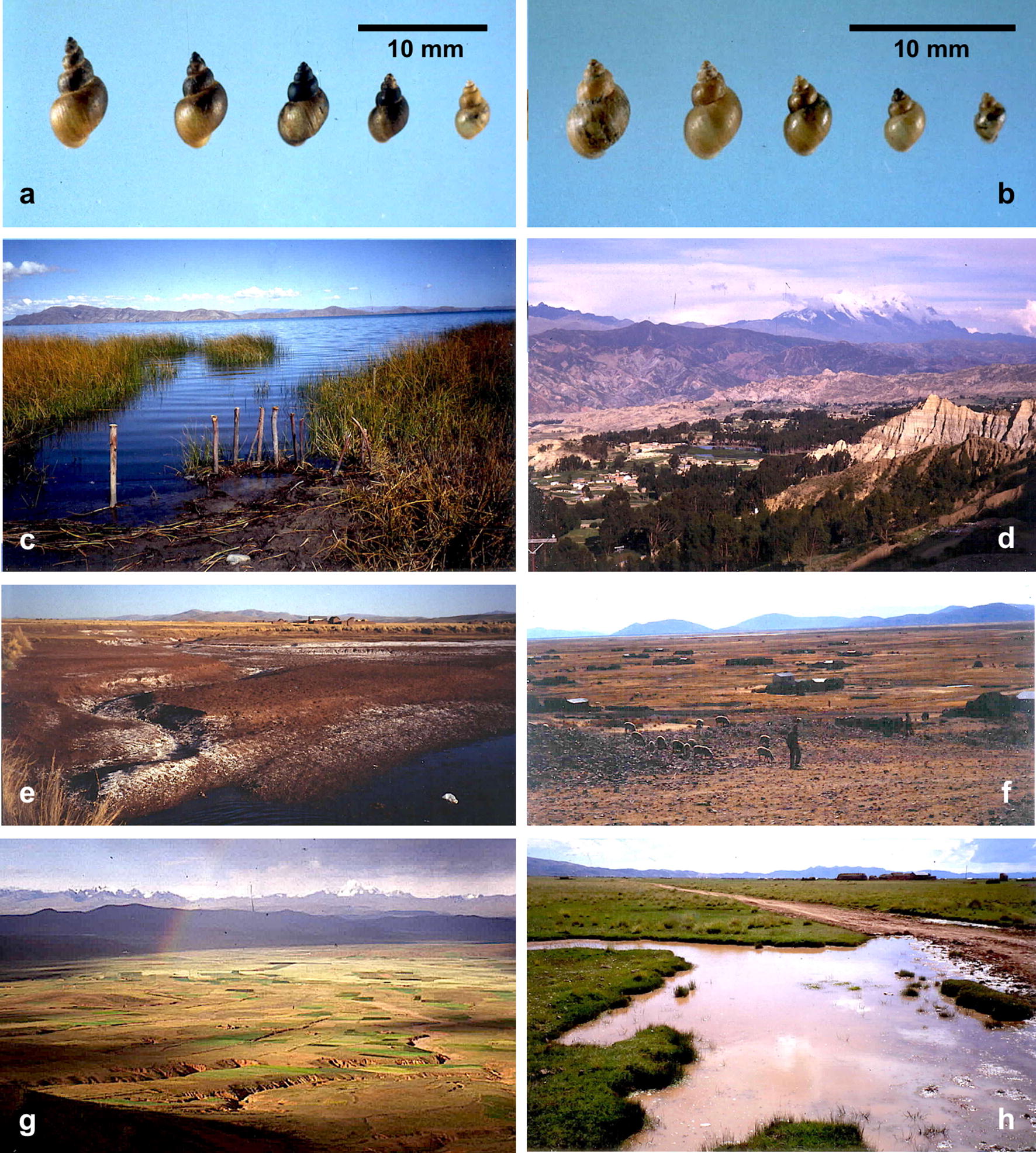
Lymnaeids, their habitats and distribution boundaries in the northern Bolivian Altiplano. **a***Galba truncatula* Morph I (*Lymnaea viatrix sensu* Ueno et al. [58]) of the northern Bolivian Altiplano. **b***Galba truncatula* Morph II (*Lymnaea cubensis sensu* Ueno et al. [58]) of the northern Bolivian Altiplano. **c** Bolivian shore of Lake Titicaca covered by typical totora (*Schoenoplectus californicus totora*). **d** Achocalla, a small fascioliasis endemic sub-valley of the large La Paz Valley. **e** Large amounts of salts on the terrestrial surface in the Catari-Capiri zone southward from Viacha. **f** Community of the Huacullani corridor showing dispersed dwellings, lymnaeid-inhabited water bodies in between and free livestock running throughout. **g** Overview of eastern part of the large corridor from Tambillo to Huacullani. **h** Subsoil effluence presenting lymnaeids close to the village of Yanarico with liver fluke infected children, in the Tiwanaku-Guaqui corridor. Photographs: SM-C

Lymnaeid shell morphology allowed to distinguish a large variability between two extreme shell forms. The shell of Morph I (Fig. 3a), when compared to the shell of Morph II (Fig. 3b), appears to be longer and only slightly wider, giving a more slender aspect. Moreover, the sutures separating the whorls are deeper and the outer striation of the shell whorls are more marked and visible in Morph I than in Morph II.

However, rDNA and mtDNA marker sequences revealed that lymnaeids from all the localities surveyed belong to a single lymnaeid species, *Galba truncatula*. Sequences reported in this paper are available in the GenBank, EMBL and DDBJ databases under the accession numbers listed in Table 1.

#### rDNA ITS2

All specimens had identical ITS2 sequence (401 bp long; GC content of 58.60%). When compared with the three complete ITS2 haplotypes described for *G. truncatula*, no nucleotide difference was found with the haplotype G.tru-H3 (GenBank: AJ272051), and previously reported in Bolivia, Chile and Argentina. Analysis of the alignment revealed that this haplotype presents a characteristic mutation at position 55, i.e. T/G in H3/H1 and H2. Moreover, H1 and H3 differ from H2 at position 149 (T/C).

#### rDNA ITS1

Similarly, all specimens showed identical ITS1 sequence (504 bp long; GC content of 57.53%). When compared with the five complete ITS1 haplotypes described for *G. truncatula*, no nucleotide difference was found with the haplotype G.tru-HC (GenBank: AJ272052) previously reported in Bolivia, Chile and Argentina. Although five nucleotide differences appear in the alignment of the five haplotypes, G.tru-HC had exclusive mutations at positions 74 and 75, allowing for the differentiation from the other four haplotypes (GT/AG in HC/HA, HB, HD).

#### mtDNA 16S

Only one haplotype was detected. This partial sequence was 425 bp long (AT content of 68.85%) and proved to be identical to the haplotype G.tru-16S-A (GenBank: HE610431) previously described for *G. truncatula* in Europe and Bolivia. Differences with haplotype G.tru-16S-B, present in Europe and in Peru, are restricted to only one mutation A/T at position 345 of the 16S-A/16S-B alignment. No complete identity was found when compared with any of the 12 variable sequences of the *16S* fragment from Argentina (GenBank: JN872477-JN872488). A total of 23 polymorphic differences appeared, including 3 indels and 20 mutations in the 432 bp long alignment of G.tru-16S-A and B with JN872477-JN872488.

#### *mtDNA* cox*1*

**Fig. 4:**
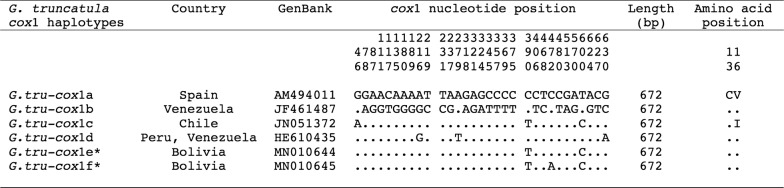
Nucleotide and amino acid differences found in the mtDNA *cox*1 sequence of *Galba truncatula* populations studied from the northern Bolivian Altiplano and other *G. truncatula* haplotypes of the same species. Position (numbers to be read in vertical) refer to variable positions obtained in the alignment made with MEGA 6.0.6 (“ . ”, identical; *, present paper). Haplotype codes are only provisional due to incomplete sequences of the gene

Two haplotypes were found, both identical in length. In all populations studied (Table 1), except in the Ayo Ayo locality, only one *cox*1 haplotype of 672 bp (AT content of 68.45%) was found. Comparisons with haplotypes so far described for this gene fragment in *G. truncatula* available on GenBank were based on a 672 bp long alignment showing a total of 32 variable positions, among which 4 parsimony informative positions and 28 singleton sites (Fig. 4). This alignment demonstrated the Bolivian haplotype to be novel, to which the code “*cox*1e” is assigned (GenBank: MN010644).

Moreover, a different haplotype was found but only in the locality of Ayo Ayo (Table 1), where it appears to be less abundant (three specimens among a total of eight sequenced) than the aforementioned dominant haplotype “*cox*1e”. This second sequence was 672 bp (AT content of 68.60%). Single nucleotide polymorphisms (SNPs) found in the 672 bp long alignment comparison are shown in Fig. 4. This sequence was characterized by one transition (G/A, at position 46) and one transversion (A/C, at position 472) when compared with “*cox*1c” and “*cox*1e”, respectively (Fig. 4). This haplotype also proved to be novel and is assigned the code “*cox*1f” (GenBank: MN010645).

The COX1 amino acid sequences of the two *cox*1 gene haplotypes from Bolivia generated only one protein haplotype, which proved to be identical to that from Spain, Venezuela and Peru (Fig. 4).

### Geographical assessment of transmission foci

#### Lymnaeid populations inside the known endemic area

The fascioliasis endemic area reaches from the west shore of Lake Titicaca (Fig. 3c) up to the valley of the La Paz city in the East (Fig. 3d). The lowest altitude of 3820 m at the Titicaca shore gradually increases northward up to the eastern Andean Chain and eastward up to El Alto city (Figs. 1d, 2a). This fascioliasis area includes several plains separated by small hill chains. These plains are locally known as corridors. The southern large extensions of land are covered by a visible superficial salt layer where lymnaeids have not been found (Fig. 3e).

The northern corridor extends from El Alto to the villages of Pucarani and Batallas up to the coast of the Lake Titicaca. It extends along that coast northward up to Achacachi and Belen, and westward up to Huatajata and Tauca. This corridor includes many freshwater habitats presenting stable lymnaeid populations (Fig. 2a). There are, however, other freshwater habitats of the so-called “bofedal” type in which lymnaeids are never present. This stability concerns both different seasons and different years. However, recent fascioliasis control measures have led to the disappearance of the local lymnaeid populations, such as the one at the old artificial fountain located in front of the Chijipata Alto school in which prevalence and intensities in children had proved to be very high.

**Fig. 5:**
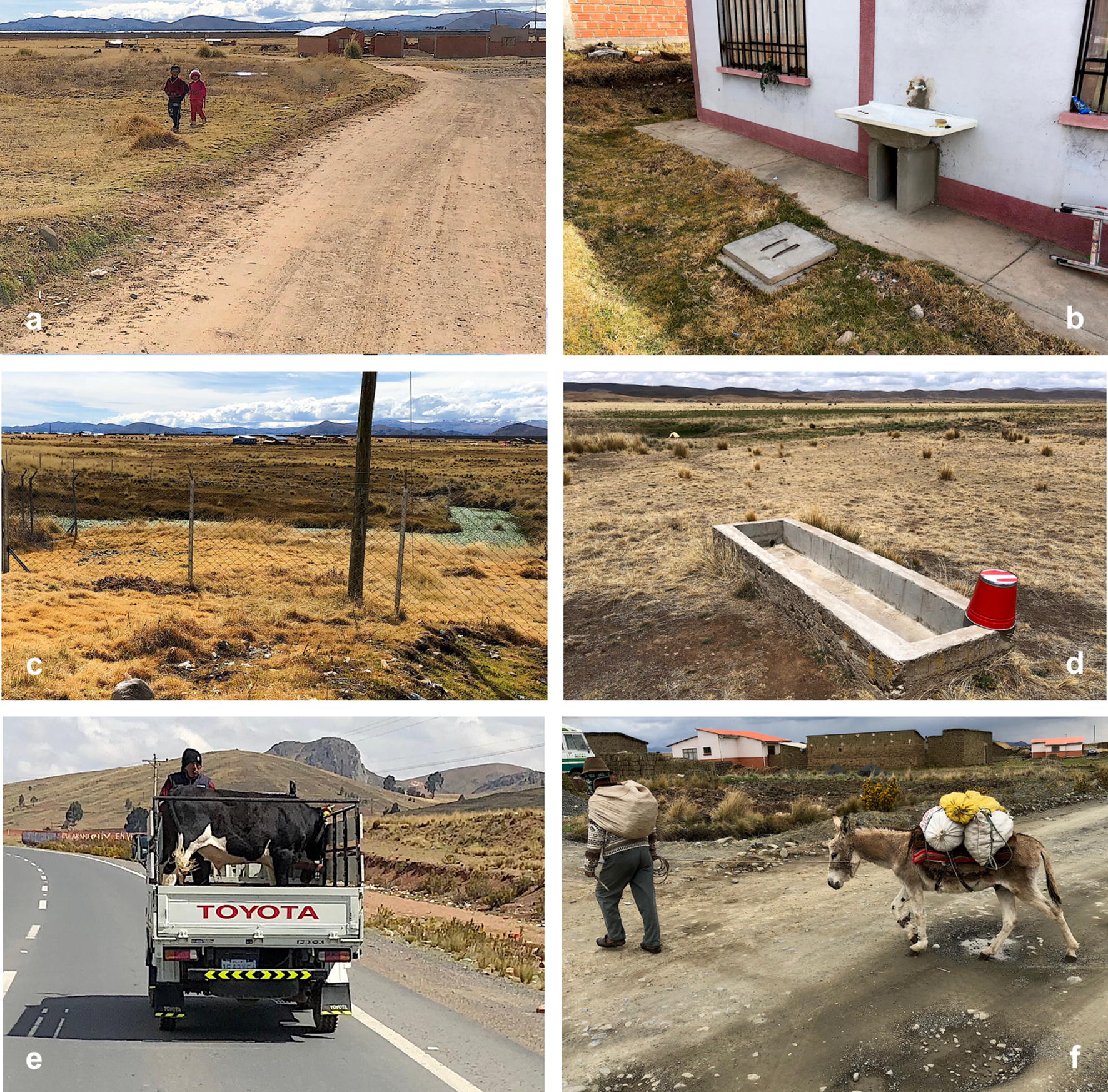
Aspects of lymnaeid control in the Northern Bolivian Altiplano. **a** Children along their daily way from home to school and back in the Huacullani zone. **b** External faucet and basin in front of a health center in Huacullani. **c** Installation of metal fences surrounding lymnaeid-inhabited water bodies close to Huacullani village. **d** Unused artificial drinking trough for livestock despite infection risk due to lymnaeid presence in neighboring river. **e** Potential large-scale lymnaeid spread due to cattle transport with trucks along the El Alto-Batallas/Peñas route. **f** Potential small-scale lymnaeid spread linked to goods and merchandise transport by donkeys in the Suriquiña zone. Photographs: SM-C

**Fig. 6:**
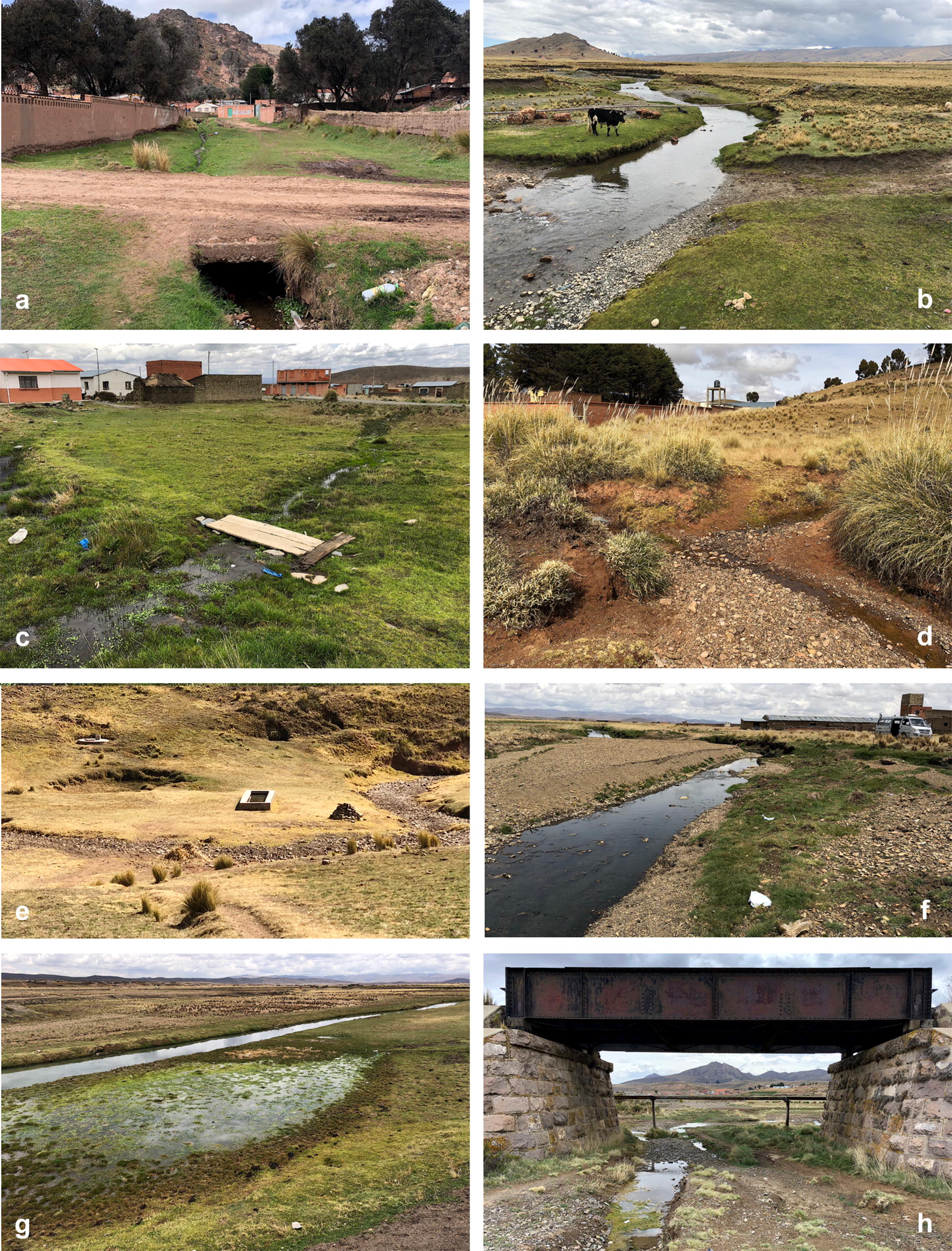
Lymnaeid freshwater habitats found outside the past-established boundaries of the human fascioliasis hypendendemic area in the northern Bolivian Altiplano. **a–c** Corridor of Peñas. **a** Peñas: small stream inside the village. **b** San Calixto: river margin close to the village. **c** Suriquiña: zone inside village flooded by stream from eastern Andean chain. **d**, **e** Rosa Pata hilly zone. **d** Rosa Pata: small stream close to school. **e** Rosa Pata surroundings: covered water well on hill side, dry stream, and lymnaeid-inhabited natural subsoil effluence preferred as water source by livestock instead of close artificial drinking trough. **f**-**h** Patacamaya zone. **f** Challapata: river margin close to rural dwellings. **g** Ayo Ayo: flooded zone next to the river under bridge on route to neighbouring village. **h** Viscachani: small stream running under train bridge with village in the background. Photographs: SM-C

The second corridor includes the villages of Laja and Tambillo and westward up to Aygachi and Huacullani (Figs. 2a, 3g). The transmission foci appear to be stable throughout this corridor. However, present road constructions are modifying the habitats where lymnaeid-inhabited freshwater habitats were found in the past (Fig. 3f). In Huacullani, despite metal fences installed to impede the access to lymnaeid-inhabited water bodies (Fig. 5c) and constructions of faucet and basin for water availability (Fig. 5b), the daily walk of children from home to school and back allows for an infection risk along the rural way (Fig. 5a). In this zone, despite the construction of artificial drinking troughs for livestock, animals continue to be infected because they prefer lymnaeid-inhabited subsoil effluences as a water source (Fig. 6e).

The third southernmost corridor extends up to Tiwanaku and Guaqui close to the Peruvian border. This corridor presents important human transmission foci, e.g. around Yanarico (Figs. 2a, 3h) and Chambi Grande.

Southward from El Alto, there are transmission foci in the surroundings of Viacha, one small focus located in the La Paz suburb of Achocalla (Fig. 3d), and several foci in the Saythu zone along the Cala Jahuira river valley (Fig. 2a).

#### Lymnaeid populations detected outside the past-established endemic boundaries

The recent surveys detected lymnaeid populations in the localities of Peñas (Fig. 6a), San Calixto (Fig. 6b) and Suriquiña (Fig. 6c). These findings represent a northward expansion along the corridor of Peñas and Kerani (Fig. 2b). Lymnaeids were never found in this corridor despite the numerous surveys in different seasons and years in the past. The construction of a wider although non-paved road may have facilitated lymnaeid arrival thanks to livestock transport with trucks (Fig. 5e). The use of donkeys for the transport of goods and merchandises may have further contributed to the snail spread at a more local level (Fig. 5f).

A second unexpected finding occurred along the route from Huacullani to Tiwanaku which crosses the hill chain separating the two corresponding corridors. Two lymnaeid populations were found in Rosa Pata, located at almost 4000 meters above sea level (masl) and 6 km far away from Huacullani, which is only at 3835 masl close to Lake Titicaca (Fig. 2c). One transmission focus is close to the primary school of the community (Fig. 6d), whereas the other focus is a subsoil effluence mainly related to livestock infection (Fig. 6e). Cattle, sheep and a few pigs were around both freshwater habitats. This is the first time that lymnaeids are found on a hill separating two corridors.

In the South, three lymnaeid populations were found along the route from El Alto to Patacamaya. The southernmost lymnaeid population was close to Viscachani, 75 km from Viacha and around 50 km from the southernmost focus known in the past in the Cala Jahuira river valley (Fig. 2d). These transmission foci were: (i) a river margin close to rural dwellings in Challapata (Fig. 6f); (ii) a flooded zone besides the river under the bridge of the route to the neighboring Ayo Ayo (Fig. 6g); and (iii) a small stream running under a train bridge close to Viscachani (Fig. 6h). These findings represent a pronounced southward spread along the north-south plain followed by the Kheto River course.

## Discussion

Veterinary surveys were the first to prove that the northern Bolivian Altiplano is a fascioliasis hyperendemic area [45, 57–62]. No infection could be found in sylvatic mammals (lagomorphs, rodents) [63]. This endemic area began, however, to attract scientific focus after the importance of the public health problem posed by this disease in children [10, 11, 31–34, 59, 64–69], including the highest prevalence and intensities ever reported in humans [10, 11, 33, 34, 59] has been shown.

The first study on the snail vectors identified two American lymnaeid species: *Lymnaea viatrix* and *L. cubensis* (Fig. 3a, b) [58]. The presence of *L. viatrix* was again reported several years later [70]. Twenty years later, another study demonstrated that they were nothing other than the extreme morphs of a large variability of *G. truncatula* [71], whose ecological characteristics in the Altiplano were assessed [72]. Subsequent phenotypic analyses by isoenzymes suggested a high monomorphism of its Altiplano populations [73]. Unfortunately, the low resolution of this phenotypic methodology did not allow for a definitive conclusion. Isoenzyme electrophoresis has shown that a very large range of situations can be found in lymnaeids, including heterogeneous, polymorphic populations [74–76]. In Peru, a sibling species complex detected by isoenzyme electrophoresis [77] could only be elucidated after DNA marker sequencing [49]. The DNA microsatellite technique, also applied to the Altiplano lymnaeids [78], is another banding method posing similar resolution limits. A parallel morphological study was useful for additional phenotypic characterization but did not provide further information [79].

DNA sequencing was applied to Morph I from the locality of Tambillo and Morph II from Batallas. The ITS2 and ITS1 sequencing confirmed they belong to *G. truncatula* [47]. The present multiple DNA sequencing showed that only one lymnaeid species inhabits the endemic area, the most efficient fascioliasis vector *G. truncatula* [30].

Consequently, this appears to be the only human fascioliasis endemic area in South America in which a single lymnaeid vector species is involved in the transmission of the disease. Many amphibious species of the *Galba*/*Fossaria* group are the main transmitters of human fascioliasis throughout the Americas [27–30, 43, 49, 50]. The lymnaeid *Pseudosuccinea columella* is mainly linked to livestock infection [29]. Three species have been reported from the hyperendemic area of Cajamarca, Peru, i.e. *G. truncatula*, *L. schirazensis* and *L. neotropica* [80], four species, *L. cousini*, *L. neotropica*, *L. cubensis* and *P. columella* have been reported in Ecuador [29], *G. truncatula*, *L. cubensis*, *L. schirazensis* and *P. columella* have been recorded to coexist in Venezuela [28], *L. neotropica* and *L. viator* in Argentina [27], and *G. truncatula* and *L. viator* in Chile [24].

A One Health initiative against fascioliasis is very complicated due to the multidisciplinary efforts needed to face the complexity of the interactions of the many organisms involved in liver fluke circulation. Differences in ecology, ethology, population dynamics, seasonality, anthropophily and fasciolid transmission capacity of each lymnaeid species define the transmission patterns and epidemiological scenarios of human and animal fascioliasis in endemic areas [8, 18, 37, 43, 81]. Thus, endemic areas presenting only one vector species show transmission and epidemiological characteristics more easily affordable, although pronouncedly differing when the lymnaeid species is different [30]. Endemic area characteristics are very complex where two or more different vector species coexist [82]. Similar interdepending relationships between disease transmission and epidemiology complexity, on one side, and the number and species of vectors, on the other side, have been described in other diseases, such as *Plasmodium* spp./*Anopheles* spp. in malaria [83] and *Trypanosoma cruzi* discrete typing units (DTUs)/Triatominae spp. in Chagas disease [84–86].

The extreme monomorphism of the four DNA sequences may be explained by a spread from an initial founder specimen in a geographical expansion phenomenon elapsed evolutionarily recently. *Galba truncatula* was introduced into South America by the Spanish ‘conquistadores’, most probably during the first centuries of the Americas colonization period [18, 47]. The initial specimen colonizing the Altiplano should have been a highly efficient vector and transmitted its high disease transmission capacity to all its descending lymnaeid generations [30, 47]. Genetic clonicity was most probably the consequence of exclusive or almost exclusive selfing (autofecundation). The usual selfing multiplication in lymnaeid species of the *Galba*/*Fossaria* group [43, 87], especially noted in *G. truncatula* [88, 89], may have been enhanced by the very high-altitude extreme conditions.

A transversion in “*cox*1f” in part of the Ayo Ayo population (Table 1, Figs. 2a, d, 4) is the only exception. Evidence suggests this mutation to have sporadically originated in this locality. The same conclusion is reached when considering that in the Patacamaya zone, Aroma Province, local infection in cattle could not be found in the past despite numerous bovine analyses in many localities [45].

In the North, the periodic floods between Lake Titicaca and the eastern Andean chain by the saline waters of the Lake Titicaca explain the absence of lymnaeids northward from Belen. The decrease of temperature with the progressive increase of altitude along the foothills of the eastern Andean chain was considered to be linked to lymnaeid absence along the northern part of the El Alto-Batallas route [34].

In the South, the boundary was established southward from Viacha [34]. The loss of the climatic moderating influence of Lake Titicaca [90, 91] is linked to low night temperatures, decrease of humidity and strong winds [92, 93], which explain the absence of lymnaeids and liver fluke. Moreover, large superficial salt extensions explain the absence of lymnaeids in this zone (Fig. 3e) [94]. This is why the transmission foci of Yanarico and Chambi Grande, isolated along the Tiwanaku-Guaqui corridor, are linked to subsoil effluences (Fig. 3h). Soil chemical composition also explained lymnaeid absence in other freshwater habitats throughout this corridor [34].

In the West, the slightly saline waters of Lake Titicaca constitute an unsurmountable boundary for lymnaeids [95–97]. Moreover, the shores of this lake are densely populated by the Cyperaceae plant called totora (*Schoenoplectus californicus tatora*) (Fig. 3c), whose root secretions have molluscicidal activity [98, 99] and further explain lymnaeid absence in the waters of Lake Titicaca [100].

In the East, temperature decrease related to altitudinal increase explains the south-eastern boundary in the Cala Jahuira River mid-valley (Fig. 2) [34].

Throughout the endemic area, *G. truncatula* is found in different types of freshwater habitats. Altiplanic habitats include small watercourses, natural and artificial canals, subsoil effluences from shallow phreatic layers, large and small rivers originating from the snow amounts of the eastern Andean chain, flooding areas, shallow wells, pools, man-made fountains, overflowings, natural clean waters, and eutrophic waters inside villages.

The comparison of the recent field results with those obtained in the 1990s [34] allows for the defining of key characteristics:

(i) *Long-term stability.* The patchy distribution of fascioliasis is linked to transmission foci which appear to be stable [34], with the exception of those modified by the construction of new roads or because of fascioliasis control measures.
(ii) *Permanent freshwater habitats.* The link of lymnaeid populations to permanent freshwater habitats is related to the high evapotranspiration rates of the high altitude [101], even despite the humidity influence of Lake Titicaca [88]. In the Altiplano, temporary water bodies originating from rainfall do not persist for sufficient time to allow colonization by lymnaeids. The few transmission foci in which water is absent during winter are habitats depending on human activities (irrigation canals, human waste in streams inside villages) or on efflorescences or streams. Consequently, the existence of lymnaeid populations along the whole year, together with the long survival of metacercariae [102], enable disease transmission during all seasons.
(iii) *Aquatic trend of lymnaeids.* Lymnaeids at Altiplano are more aquatic than their markedly amphibious conspecific European populations. This facilitates infection by the swimming miracidium and higher snail population infection rates underlaying the high disease transmission rates [30, 47]. However, recent lymnaeid findings on mud, out of water, suggest an increasing trend to amphibiousness, at least in certain localities.
(iv) *Absence of shade.* In the Altiplano, there is almost no shade due to the lack of trees and shrubs. Thus, the intense sunshine of the high altitude [90, 103] directly falls upon the water bodies. This facilitates the growth of freshwater algae on which lymnaeids mainly feed, moreover, it increases the temperature of water bodies at midday when lymnaeids have maximum activity, an important factor considering the very low night air temperature at high altitude [101]. It should be remembered that the northern Altiplano was originally a forest [104]. Today only a few trees remain, including willows and eucalyptus in only a few places, and a very few endemic kishuara *Buddleya coriacea*. This man-made deforestation may have facilitated the spread of lymnaeids throughout the endemic area.

Three unexpected findings in the present field surveys should be highlighted because they demonstrate a present phenomenon of geographical expansion of the fascioliasis transmission risk:

(i) *A northward spread.* The finding of *G. truncatula* in three localities of the northern corridor of Peñas-Kerani indicates a septentrional spread (Fig. 2b). In spite of the exhaustive field surveys carried out along this corridor in the 1990s, lymnaeids were never found [34], neither was liver fluke infection detected in cattle [45] nor in humans [33]. A study of the distribution of the disease in the Altiplano by means of remote sensing (RS) tools (NDVI index) indicated that this corridor was nevertheless *a priori* suitable for fascioliasis transmission [105]. This suggests a recent northward expansion, probably related to lymnaeid introduction with livestock imported from other zones of the endemic area. According to previous research, lymnaeids may remain in dried mud stuck to the feet of ruminants, then go into hibernation or estivation, and are able to reactivate once in a new location following contact with water or sufficient humidity [18]. The recent road improvement in 2018 may have facilitated such livestock transport. In personal interviews, Aymara inhabitants of this zone told us about liver fluke infection in local livestock and the treatments they implement against this infection.
(ii) *An altitudinal spread.* Lymnaeid populations had never been found on hill chains separating corridors. This absence was thought to be linked to their altitude, with temperatures too low during the night [34]. The discovery in the present study of two lymnaeid populations on the hill chain separating the Tambillo-Huacullani corridor and the Tiwanaku-Guaqui corridor was unexpected. The altitude of the site in which snails were found (Rosa Pata, at 4965 masl), is higher than that of all transmission foci found in the Altiplano in the past (Table 1, Fig. 2c). Interestingly, this altitude falls within the altitudinal range of the new transmission foci detected in the Peñas-Querani corridor (Peñas: 3986 masl; San Calixto: 3970 masl; Suriquiña: 4001 masl) (Table 1, Fig. 2b). This suggests that the lack of transmission foci in this northern corridor in the past may have been also due to excessive altitude.
(iii) *A southward spread.* The large north-south plain along the route from El Alto to the Central Altiplano was, in the past, lacking fascioliasis transmission risk [34, 42]. The low night temperatures due to the loss of the temperating influence of the distant Lake Titicaca were considered to be the cause of the absence in the Central Altiplano [34]. The present discovery of *G. truncatula* in this zone means a southward spread of 75 km and 50 km regarding the past southernmost transmission foci in Viacha and Cala Jahuira river valley, respectively (Fig. 2d). Moreover, autochthonous livestock owners of this area told us about their livestock being infected by “Talpalako” (Aymara name for liver fluke) and the fascioliasis treatments they apply.

The three aforementioned phenomena of geographical expansion suggest potential effects of global warming. Increasing temperatures may be transforming previously altitude-unsuitable areas to suitable transmission areas and consequently allowing for arrival and colonization by lymnaeids.

The endemic area proves to change over time, including potential changes in the inner endemic foci due to human activities and potential outer spread due to the influence of climate change. This means that the distribution of transmission foci will need to be re-assessed from time to time in the future.

## Conclusions

The following conclusions may be reached regarding the extension of the local One Health initiative to the whole human hyperendemic area of the Altiplano. An area where the disease is caused by only one *Fasciola* species and transmitted by only one lymnaeid vector species is a great advantage for control because of the less complex efforts needed. The molecular monomorphism of the lymnaeid populations suggests an additional simplification. Accordingly, uniformity of responses by all lymnaeid populations is expected after implementation of the control measures. The local differences of *G. truncatula* population dynamics linked to seasonal variations of the habitat microclimate [106] are expected to have no control repercussions, although appropriate studies are still pending. The instability of the outer boundaries of the fascioliasis transmission risk area suggests an impact of climate change whose further influencing trend should be assessed by appropriate tools. A global warming phenomenon may explain the three areas of lymnaeid spread detected. All lymnaeid populations discovered outside the previously known boundaries of the hyperendemic area are close to villages, human dwellings and/or schools. Consequently, control activities should henceforth include the new zones for preventive chemotherapy implementation. The findings of lymnaeid populations in the Patacamaya zone concern a fifth province which was never considered before. Therefore, the Province of Aroma should henceforth be included within the preventive chemotherapy strategy. Regarding a One Health action, this study highlights the need for: (i) a previous verification of the lymnaeid vector species involved; (ii) assessment of the genetic variability of its (or their) populations by appropriate multiple DNA marker sequencing; (iii) periodic monitoring of the stability of the transmission foci inside the known geographical distribution of the endemic area; and (iv) field surveys covering zones outside this area to assess a potential lymnaeid vector spread due to either human activities, such as irrigation systems [12], livestock movements [18], or climate change effects [15, 107].

## Data Availability

Data supporting the conclusions of this article are included within the article. The newly generated mtDNA *cox*1 haplotype sequences were submitted to the GenBank database under the accession numbers MN010644 and MN010645.

## Abbreviations

ITS2: Second transcribed spacer of the nuclear ribosomal DNA operon
ITS1: First transcribed spacer of the nuclear ribosomal DNA operon
*16S*: Ribosomal RNA large subunit gene of the mitochondrial DNA
*cox*1: Cytochrome *c* oxidase subunit 1 gene of the mitochondrial DNA
